# Special Issue: Recent Research on Hypertension and Related Complications

**DOI:** 10.3390/ijms27136031

**Published:** 2026-07-05

**Authors:** Charlotte Delrue, Marijn M. Speeckaert

**Affiliations:** 1 Department of Nephrology, Ghent University Hospital, 9000 Ghent, Belgium; charlotte.delrue@ugent.be; 2 Research Foundation-Flanders (FWO), 1000 Brussels, Belgium

Hypertension is still the most important risk factor in which alterations can control heart and kidney diseases, stroke, and early deaths worldwide. Although there have been many improvements in the prevention, detection, and drug treatment of high blood pressure for several decades, more than one billion people around the world are still affected by it, with hypertension responsible for a large number of disability-adjusted life years and health expenses. Great strides have been made in reducing the risk of heart disease through blood pressure drugs; however, their application remains rare across many populations, indicating gaps in awareness, diagnosis, treatment adherence, and implementation of evidence-based care [1,2,3]. The ongoing worldwide problem of high blood pressure is not only due to its widespread prevalence, but also its extremely diverse and complex set of underlying causes. Increasingly, research is showing that simple blood vessel resistance or salt retention disorders cannot fully explain high blood pressure. On the contrary, it should be viewed as a multifactorial, biologically heterogeneous syndrome resulting from the interplay of genetic susceptibility, environmental factors, neurohumoral regulation, immune activation, metabolic dysfunction, vascular remodeling, and target-organ vulnerability [4,5,6,7,8,9].

The transition from a hemodynamic model to a systems biology perspective represents perhaps the most significant conceptual evolution in hypertension research over the last ten years [10,11,12,13]. Hypertension was previously mainly considered an outcome of abnormalities in renal sodium handling, sympathetic activation, and the renin–angiotensin–aldosterone system (RAAS). Although these mechanisms still play a role in blood pressure regulation, new research has uncovered a much broader range of biological processes involved in disease initiation, progression, and complications. The development of high-throughput technologies, such as genomics, transcriptomics, proteomics, metabolomics, and single-cell sequencing, has revolutionized our understanding of hypertension as a single clinical diagnosis [14,15]. Today, it is viewed as the result of a group of biologically distinct disease entities characterized by distinct molecular pathways and therapeutic responses [16,17,18].

The articles in this Special Issue highlight these changes. Together, the nine papers explore new ways of regulating blood pressure, the causes of target-organ injury, biomarker discovery, vascular changes, resistant hypertension, and innovative therapeutic approaches. Most of the studies address three main changes that are currently transforming the field: the shifts from hemodynamics to systems biology, clinical phenotypes to molecular endotypes, and standard antihypertensive treatment to mechanism-based precision therapeutics [19,20,21,22,23,24,25,26,27] (Figure 1).

Growing awareness of neglected neurohumoral pathways highlights the rapid expansion of our biological understanding of hypertension. Lee et al. [19] conduct an extensive review of the role of neuropeptide FF (NPFF) and its receptors in central and renal blood pressure regulation. Beyond the well-known RAAS and sympathetic nervous system, NPFF signaling appears to influence autonomic regulation, sodium handling, and cardiovascular stability through coordinated actions in the hypothalamus, brainstem, and kidneys [28,29]. These findings support a new view in which hypertension results from impaired interactions among brain–kidney networks rather than isolated organ dysfunction. NPFF receptors as new drug targets underscore the growing importance of neuropeptide science in heart disease and emphasize the need to examine regulatory systems beyond those that are established.

The broader societal and epidemiological aspects of hypertension are equally critical. Goorani et al. [20] highlight that hypertension remains one of the most critical public health issues globally. Factors such as rapid urbanization, aging populations, rising obesity, physical inactivity, high dietary sodium intake, and social inequalities have together led to the worldwide prevalence of hypertension and its complications. Moreover, control rates remain low even with effective pharmacological agents, indicating that biological advances alone will not suffice. Going forward, a combination of public health measures, digital healthcare, personalized risk assessment, and population-based preventive approaches will be needed to address not only the biological aspects but also the societal factors behind hypertension.

Among the target organs affected by hypertension, the kidney holds a uniquely central position, functioning as both a mediator and victim of disease progression. Classical models emphasized glomerular hypertension, abnormalities in pressure natriuresis, and nephrosclerosis as primary mechanisms of hypertensive kidney injury [30]. However, recent discoveries have revealed a substantially more complex pathophysiological landscape [31,32]. Delrue and Speeckaert [21] comprehensively summarize how chronic RAAS activation, oxidative stress, endothelial dysfunction, mitochondrial injury, metabolic reprogramming, epigenetic alterations, and immune-mediated inflammation collectively contribute to hypertensive kidney disease. The emerging concept of prehypertensive kidney injury is crucial, suggesting that molecular and cellular abnormalities may develop before clinically detectable elevations in blood pressure. This observation challenges traditional disease models and raises the possibility that early molecular intervention could prevent irreversible target-organ damage long before hypertension clinically manifests.

Delrue and Speeckaert’s article also points to another significant change in cardiovascular medicine: the rise of precision nephrology [21]. This area of medicine, thanks to transcriptomic profiling, urinary proteomics, plasma metabolomics, spatial biology, and single-cell RNA sequencing, is uncovering molecular disease subtypes in ever-greater detail [33,34,35,36,37,38]. Instead of simply assigning patients to blood pressure categories, we could, with the help of these new technologies, identify biologically distinct endotypes characterized by distinct inflammatory, fibrotic, metabolic, or vascular signatures [39,40,41,42,43]. Molecular stratification of this kind can pave the way for personalized therapy selection and more accurate disease progression prediction. Changes like this are occurring throughout cardiovascular medicine and will likely change hypertension management over the next 50 years [16,17].

Recognizing that immune system imbalance and chronic inflammation not only result from high blood pressure but also actively drive the development of the disease is perhaps one of the biggest breakthroughs in today’s hypertension research. Through laboratory work, different types of immune cells and pathways, such as activated T cells, infiltrating macrophages, interleukin-17 (IL-17) production, NOD-, LRR-, and pyrin domain-containing protein 3 (NLRP3) inflammasome activation, oxidative stress, and neuroimmune communication, have been shown to lead to hypertension and organ damage. These inflammatory cascades cause endothelial dysfunction, changes in blood vessel structure, kidney damage, fibrosis, and sustained increases in blood pressure. In fact, the concept of inflammation offers a coherent biological explanation for various forms of hypertension that appear different at first glance. Inflammatory and immune mechanisms are found not only in obesity-related hypertension and hypertensive kidney disease but also in resistant hypertension, pregnancy-induced hypertension, and vascular remodeling. This new concept will likely lead to the development of new therapeutics targeting the immune system in the near future [6,7,8,9,44,45,46].

The role of inflammation is highlighted in several articles in this Special Issue. Sosnicka et al. [27] reveal the linkages between blood pressure and circulating levels of leptin, interleukin-6 (IL-6), and vascular endothelial growth factor (VEGF) in subjects with childhood and adolescent obesity. Their data strengthen the idea that fat tissue is not only a site of fat storage but also an endocrine and immunological organ that can affect sympathetic activation, endothelial function, insulin resistance, and vascular inflammation. The discovery of these markers underscores the role of immunometabolism in hypertension among children and points to the necessity of early cardiovascular risk identification in children with obesity. In fact, with the growing epidemic of childhood obesity worldwide, biomarker-based risk stratification methods may become key in preventive cardiovascular medicine [47,48].

A second major transition shaping hypertension research is the shift from conventional clinical phenotyping to molecular phenotyping and precision medicine. For a long time, hypertension management was largely based on blood pressure measurements taken during office visits, alongside classic cardiovascular risk factors. However, substantial differences in disease progression, target-organ vulnerability, and therapeutic response remain. As a result, the search for biomarkers that can enhance risk prediction and help tailor treatment to individuals is gaining momentum [16,49]. The study by Fernandez-Castro et al. [22] provides an elegant example of this approach by integrating ambulatory blood pressure monitoring with serum uric acid and angiogenesis-related biomarkers to predict uncomplicated hypertension during pregnancy. Their findings demonstrate that nighttime blood pressure indices, combined with a composite uric acid–angiogenic index, significantly improve the prediction of hypertensive complications. Such approaches illustrate how future risk stratification may increasingly combine physiological measurements, molecular biomarkers, and computational modeling to achieve more precise prediction and intervention.

Molecular phenotyping is not only about finding a biomarker. It is the latest frontier in genomics, polygenic risk scoring, machine learning (ML) and artificial intelligence (AI) [50,51]. Hypertension therapy, for instance, could be based primarily on multi-omics techniques that identify biologically distinct disease subtypes even before symptoms become visible. In this way, targeted upstream prevention, improved drug selection, and more effective treatment responses through monitoring changes could be achieved. Combining wearable devices, digital health tools, and AI-based algorithms for prognosis could be a key driver of precision cardiovascular medicine [16,17,18].

Precision phenotyping aims to characterize disease heterogeneity more accurately. However, much research focuses on the pathological mechanisms that lead to target-organs injury. Among these, vascular remodeling stands out as a key factor. Although pulmonary hypertension is fundamentally different from systemic arterial hypertension, the two share mechanisms, including endothelial dysfunction, inflammation, oxidative stress, fibrosis, and abnormal vascular cell proliferation. In their review, Zhang et al. [25] present evidence that flavonoids may serve as therapeutic agents for treating pulmonary arterial remodeling. These naturally occurring compounds can simultaneously affect multiple biological pathways, including oxidative stress, inflammatory signaling, endothelial injury, and cellular proliferation [52]. These findings are also highly relevant to systemic hypertension, where vascular remodeling plays a similar role in disease progression and organ damage. As more biological mechanisms shared across cardiovascular diseases are identified, therapies may be developed to target common pathogenic pathways rather than isolated disease manifestations.

Resistant hypertension is another powerful example of how biological factors contribute to blood pressure dysregulation [53,54]. While it has mainly been seen as a major therapeutic hurdle, resistant hypertension is now considered a separate clinical phenotype, hallmarked by genetic predisposition, neurohumoral activation, inflammation, renal dysfunction, and vascular remodeling [5]. Nguyen et al. [26] provide a thorough review of resistant hypertension in patients with intracerebral hemorrhage, a scenario in which controlling blood pressure effectively is crucial to limiting hematoma expansion and improving recovery. In addition to discussing standard approaches, the authors explore emerging mechanisms, including ferroptosis, neuroinflammation, and the gut–brain axis. Their discoveries reflect a broader movement toward understanding hypertension as an outcome of interactions among organs and complex biological networks. Resistant hypertension may be a beacon for future precision medicine, as it demonstrates that similar clinical manifestations can stem from distinct biological processes, underscoring the need for targeted therapies.

A third major change raised by this Special Issue concerns therapeutic innovation. Several decades ago, antihypertensive treatment relied largely on a small number of drug classes targeting the sympathetic nervous system, RAAS, calcium signaling, and renal sodium handling. These medications are still very effective; yet, they do not sufficiently account for the biological diversity of hypertension and often fail to achieve better blood pressure control among high-risk individuals. As a result, much research focuses on developing drugs that target new molecular pathways [18,45,55,56].

One of the most exciting recent developments is the emergence of RNA interference-based therapeutics. Morosan et al. [23] review the rapidly expanding evidence on zilebesiran, a small interfering RNA designed to suppress hepatic angiotensinogen synthesis. By directly targeting the source of RAAS activation, zilebesiran offers a fundamentally different therapeutic approach from conventional angiotensin-converting enzyme (ACE) inhibitors and angiotensin receptor blockers (ARBs). Clinical studies have demonstrated substantial, durable reductions in blood pressure that persist for several months after a single administration. Such long-acting therapies may significantly improve adherence while providing sustained cardiovascular and renal protection. More broadly, zilebesiran represents a landmark example of how advances in molecular medicine are beginning to transform cardiovascular therapeutics [23,57,58].

Another very interesting development is the rekindled enthusiasm for endothelin biology. In their review, Bank-Mikkelsen et al. [24] summarize recent findings on a drug called aprocitentan, which acts via the dual inhibition of endothelin receptors and is considered a means of blocking one of the body’s strongest vasoconstrictive systems. In resistant hypertension, pathophysiological features are mediated by endothelin signaling, including vascular remodeling, sodium retention, endothelial dysfunction, and inflammation, and clinical trials have shown that patients who have not responded well to standard therapies can achieve significant reductions in blood pressure with aprocitentan. The development and approval of aprocitentan highlight that re-examining known biological pathways using more effective pharmacological agents can lead to major therapeutic breakthroughs [24,59].

These innovations should be seen as part of a broader picture of the ongoing revolutionary changes in hypertension treatment [60]. In addition to RNA interference and endothelin receptor antagonism, new approaches to hypertension treatment also include aldosterone synthase inhibitors, non-steroidal mineralocorticoid receptor antagonists, anti-inflammatory therapies, gut microbiome modulation, device-based interventions, and renal denervation. Additional innovations include selective aldosterone synthase inhibitors such as baxdrostat and lorundrostat, which directly target aldosterone biosynthesis and have demonstrated promising blood pressure-lowering effects in resistant hypertension. Together with non-steroidal mineralocorticoid receptor antagonists and catheter-based renal denervation, these therapies illustrate the increasing diversity of mechanism-specific interventions entering clinical practice. Together, these treatment methods point to a departure from a sole focus on lowering blood pressure toward interventions based on biological mechanisms aligned with specific pathways and patient features [16,18,45].

In conclusion, the articles in this Special Issue collectively demonstrate a remarkable shift in hypertension research. Rather than focusing predominantly on reductionist models of hemodynamics, the field is gradually shifting toward integrated approaches that span immunology, metabolism, systems biology, molecular phenotyping, and precision therapeutics. A growing body of research indicates that hypertension is not merely a single disease but rather a collection of distinct disorders, each with its own biological features, molecular signatures, target organs, and therapeutic efficacies. Ongoing advances in multi-omics technologies, AI, biomarker discovery, and therapeutic development are sure to accelerate the field’s transition [61].

As a result, the articles in this Special Issue provide a good representation of the current key trends and issues in hypertension research. They cover many topics, including new neurohumoral pathways, public health issues, personalized kidney treatment, biomarker-based risk assessment, vascular changes, difficult-to-control hypertension, and new treatment approaches, and show the great diversity and high level of activity in this field. They reveal a moment in hypertension treatment when knowledge of the biological basis of an indidvidual’s presentation will have the greatest influence, rather than clinical figures alone. The convergence of biological systems, molecular-level medicine, and tailor-made treatments is a once-in-a-lifetime opportunity to more effectively address prevention, diagnosis, and therapy, thereby significantly reducing the worldwide incidence of hypertension and its complications. Going forward, the challenge will no longer be simply lowering blood pressure, but identifying the biological mechanisms responsible for hypertension in each individual patient and selecting therapies that specifically target those mechanisms.

## Figures and Tables

**Figure 1:**
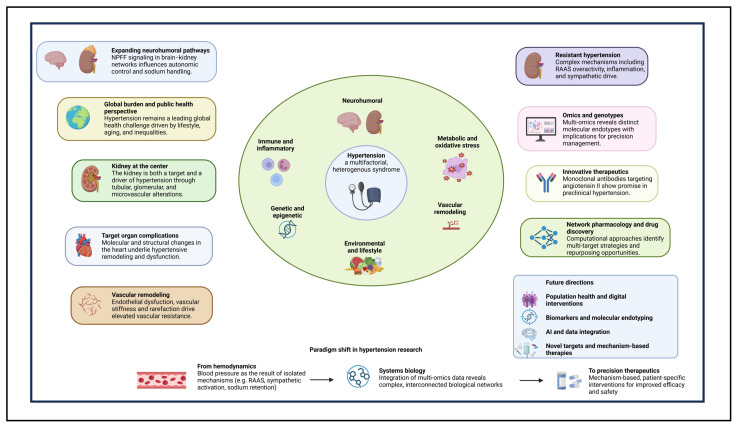
Contemporary conceptual framework of hypertension: from systems biology to precision medicine [13,14,15,16,17,18,19,20,21]. This diagram highlights the key topics of recent advances in hypertension research featured in this Special Issue. Today, hypertension is increasingly viewed as a complex syndrome composed of distinct biological types, with an emphasis on interactions among neurohumoral, immune–inflammatory, metabolic, genetic, vascular, environmental, and renal pathways, rather than as a simple problem of blood pressure regulation. Key factors include dysregulation of brain–kidney communication pathways, immune system activation, oxidative stress, vascular changes, (epi)genetic vulnerability, and lifestyle-related factors. The diagram illustrates the shift in thinking underway, moving away from traditional hemodynamic models toward systems biology approaches that integrate multi-omics technologies, biomarker discovery, and molecular endotyping. These advances enable a transition from traditional management, which is largely based on patient phenotype, to precision medicine strategies that identify biologically distinct disease subtypes and provide targeted treatment. New therapies include RNA interference-based approaches, endothelin receptor antagonism, immune-targeted interventions, and other mechanism-based treatments. Future plans include the use of artificial intelligence, digital health technologies, molecular biomarkers, and personalized risk stratification to improve the prevention, diagnosis, and treatment of hypertension and related target-organ complications. In a nutshell, the illustration captures the main idea of this Special Issue: that the use of systems biology and precision medicine is crucial to curbing the global problem of hypertension and its cardiovascular and renal consequences.

## Abbreviations

The following abbreviations are used in this manuscript:

ACE	Angiotensin-Converting Enzyme
AI	Artificial Intelligence
ARB	Angiotensin Receptor Blocker
IL	Interleukin
ML	Machine Learning
NLRP3	NOD-, LRR-, and Pyrin Domain-Containing Protein 3
NPFF	Neuropeptide FF
RAAS	Renin–Angiotensin–Aldosterone System
RNA	Ribonucleic Acid
siRNA	Small Interfering RNA
VEGF	Vascular Endothelial Growth Factor
